# Mechanistic insights into encapsulation and release of drugs in colloidal niosomal systems: biophysical aspects†

**DOI:** 10.1039/d1ra06057k

**Published:** 2021-10-29

**Authors:** Eva Judy, Manu Lopus, Nand Kishore

**Affiliations:** Department of Chemistry, Indian Institute of Technology Bombay Powai Mumbai 400 076 India nandk@iitb.ac.in; School of Biological Sciences, UM-DAE Centre for Excellence in Basic Sciences, University of Mumbai Vidyanagari Mumbai 400 098 India

## Abstract

Vesicular systems such as niosomes provide an alternative to improve drug delivery systems. The efficiency of a drug delivery vehicle is strongly dependent on its components which decide its interaction with partitioned drug(s) and locus of site of partitioning. A quantitative understanding of the physical chemistry underlying partitioning of drugs in complex systems of self-assemblies such as niosomes is scarcely available. In order to obtain quantitative mechanistic insights into partitioning and release of drugs [mitoxantrone (MTX) and ketoprofen (KTP)] in systems of niosomes, we have employed ultrasensitive calorimetry, spectroscopy and microscopy to establish correlations between functionality and energetics which could provide guidance towards rational drug design and choice of suitable non-ionic surfactant-based drug delivery vehicles. Electron microscopy and dynamic light scattering (DLS) methods were used for characterization and assessing the morphology of niosomes. We present here a calorimetry-based approach in assessing the partitioning of the anticancer drugs mitoxantrone and ketoprofen in niosomes and their release to human serum albumin (HSA) employing isothermal titration calorimetry (ITC), differential scanning calorimetry (DSC) and comparison with equilibrium dialysis. The thermodynamic signatures and kinetics of release were analyzed to obtain insights into the role of the functional groups on the drugs in the partitioning process. The assessment of thermal and conformational stability of proteins during drug binding and the effect of drug delivery vehicles on proteins is also crucial. To assess these effects, DSC studies on HSA in the presence and absence of drugs and niosomes were also performed. Finally, the efficacy of the system to impact the cell viability of the MDA-MB-231 triple-negative breast carcinoma cell line was analysed using MTT assay.

## Abstract 2

Vesicular systems such as niosomes provide an alternative to improve drug delivery systems.

## Graphical abstract

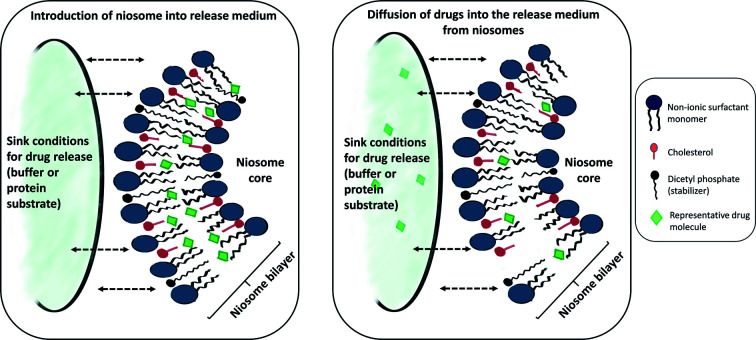

## 1. Introduction

Drugs can be administered through oral, mucosal, transdermal or transepithelial pathways in the form of pills, capsules, ointments or injectables, or encapsulated in drug delivery systems (DDS).^1–6^ To overcome problems of solubility and adequate distribution, novel drug delivery carriers have been developed, such as niosomes, liposomes, microspheres, nanoparticles and microemulsions.^7–11^ An efficient drug delivery vehicle should be biocompatible, possess high encapsulation efficiency, and show characteristics of site-specific and controlled drug delivery with negligible leaching of drugs through the carrier.^12–14^

Though liposomes having biodegradable characteristics have been explored extensively as drug delivery systems, their low stability in aqueous media limits their applicability.^12,13,15^ This has led to searches for alternate amphiphilic molecules which can form vesicular carriers. Although structurally similar to liposomes, niosomes have been reported to provide several advantages such as target-oriented drug delivery, increased transdermal drug delivery, and novel materials for drug delivery applications.^16–18^ Niosomes are macroscopic lamellar structures, generally unilamellar or multilamellar, based on their preparation method.^19^ Being amphiphilic, niosomes can encapsulate hydrophilic drugs in the core, and hydrophobic drugs within the bilayers. They can thus provide efficient encapsulation of both hydrophobic and hydrophilic drugs.^20,21^ Research efforts on niosomes have increased in the past decade due to their resemblance to liposomes and added advantage of increased physical and chemical stability in aqueous environments.^22–24^

The efficiency and drug delivery characteristics of niosomes is dependent strongly on the components that lead to their formation. The components of niosomes include non-ionic surfactants, a hydration medium, stabilizers and lipids (cholesterol). These additives are important in providing versatility,^25^ varied physical properties,^26^ mechanical strength and entrapment efficiency of niosomes.^26,27^ Further, niosomes with smaller size show enhanced permeation through inflamed and tumour cells with leaky vasculature and hence are used for target-oriented drug delivery.^11^

In spite of several such studies, the information on niosomes based drug delivery systems has remained qualitative in nature. Studies on effect of drug molecular weight on niosome size and encapsulation efficiency,^28^ and optimization of the process parameters for better encapsulation of drugs in niosomes^29^ have also enabled qualitative understanding. Loh *et al.*^30^ reviewed the use isothermal titration calorimetry (ITC) in understanding association of surfactants with polymers quantitatively, which is important in obtaining mechanistic insights into interactions. However, quantitative correlations of properties of self-assemblies with those of drugs being encapsulated in terms of their functionalities so far has mainly been limited to simple micelles.^31–34^ Such correlations are expected to guide selection of suitable systems in developing target-oriented drug delivery vehicles. Specifically, energetics of interactions provide role of functionality in meeting these targets. As a further step in understanding encapsulation and release properties of drugs in more complex but effective drug delivery systems, in this work we have used niosomes. Span 60 (sorbitan monostearate) has been used to prepare niosomes due to their low hydrophilic–lipophilic balance and high encapsulation efficiency, leading to the formation of small unilamellar vesicles ranging from 50 to 200 nm. Niosomes were also prepared encapsulating drugs (anti-cancer drug, mitoxantrone and anti-inflammatory drug, ketoprofen) to assess their target-oriented delivery.

The choice of suitable drug delivery systems requires a thorough understanding of role of functionality on both the drug as well as the delivery vehicle. To obtain quantitative insights into partitioning and release of drugs in niosomes, we have employed ultrasensitive calorimetry, spectroscopy and microscopy to establish structure–function–energetics relationships. A new experimental protocol is explored using ITC and monitoring interaction of the released drug from niosomes with transport protein human serum albumin (HSA). The thermodynamic signatures and kinetics of release were analyzed to obtain insights into the role of functional groups present on drugs in the partitioning process. The assessment of thermal and conformational stability of protein during drug binding and the effect of drug delivery vehicles on its structural integrity is also crucial. Differential scanning calorimetric studies of HSA in the presence and absence of drugs and niosomes were also performed to assess changes in thermal and conformational stability the protein. The effect of the vesicular assembly and drug release from niosomes was also assessed on MDA-MB-231 breast cancer cells to check viability of the systems *in vitro*. A comprehensive quantitative and qualitative study has been done on the formation and applicability of non-ionic vesicular systems for effective drug delivery of the chosen anti-cancer and anti-inflammatory drugs.

## 2. Materials and methods

### 2.1. Materials

**Fig. 1:**
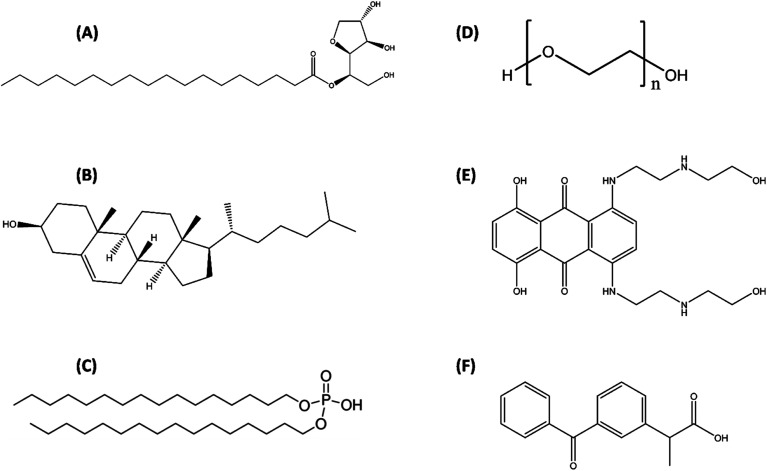
Structures of components used for preparation of niosomes (A) sorbitan monostearate (Span 60), (B) cholesterol, (C) dihexadecyl phosphate, (D) polyethylene glycol and drugs incorporated in niosomes (E) mitoxantrone (MTX) and (F) ketoprofen (KTP).

Sorbitan monostearate (Span 60 abbreviated here as S60 (>98%)) was purchased from TCI Chemicals, Japan. Human serum albumin (HSA, (>97%)), dicetyl phosphate (>97%), disodium hydrogen phosphate (>99%), sodium chloride (>98%), potassium chloride (>99.5%), potassium dihydrogen phosphate (>99%), mitoxantrone (MTX, (>97%)) and ketoprofen (KTP, (>98%)) were procured from Sigma Aldrich Co. USA, whereas cholesterol (>99%), polyethylene glycol (PEG 4000, (>95%)) and solvents (chloroform and methanol (>99.8%)) were purchased from SRL Pvt. Ltd. India (Fig. 1). The hydration of niosomes and preparation of stock solutions of the drugs and protein were done in 20 mM phosphate buffer saline (PBS) at pH 7.4. The protein solutions were subjected to extensive dialysis at 4 °C with a minimum of three changes followed by determining its concentration on a Jasco V-550 double beam spectrophotometer using an extinction coefficient corresponding to *A*_280_^1%^ = 5.8.^35^ The weight measurements were done on a Sartorius BP 211D digital weighing balance with a readability of 0.01 mg.

### 2.2. Niosome preparation

The niosomes were prepared by the thin-film hydration method. In the first step, S60 and cholesterol were dissolved in a 1 : 1 mol% ratio, followed by a negative stabilizer, hexadecyl phosphate, added in the ratio of 0.05 mol%. The concentration of S60 in the niosomes was maintained at 10 mM, which is higher than its CMC.^36^ In the formulations involving PEG, 1 mol% of the polymer was added to the vesicles in the organic phase. The components were dissolved in chloroform: methanol mixture in a ratio of 9 : 1 v/v and then evaporated using a rotary evaporator. The second step involved the hydration of the thin layer of pre-niosomes using PBS. Initially, the components were hydrated using PBS at 60 °C for 30 minutes at a continuous stirring rate of 250 rpm, followed by hydration at room temperature for a minimum period of 24 hours. This hydration time and a constant stirring rate were maintained for all the niosomes under preparation.

The drugs encapsulation was done during the second step of niosome preparation. The drugs MTX (1 mM) and KTP (5 mM) were added to the PBS buffer during rehydration and incorporated in the pre-niosomes. During hydration, depending on hydrophobic character, the drugs partition in the layers of the vesicles. The niosomes (in the absence and presence of drugs) were then subjected to vortexing and sonication for 30 min each to obtain vesicles of uniform size.^37,38^ The excess drugs were removed by dialysis before being subjected to analysis by biophysical methods. Span 60 niosomes and Span 60 with PEG niosomes were prepared, abbreviated as S60 and S60-PEG. The drug encapsulated systems were abbreviated as S60 + MTX, S60 + KTP, S60-PEG + MTX and S60-PEG + KTP. After preparation, the vesicles (in the presence and absence of drugs) were stored at 4 °C and used within ten days to avoid the complexities related to vesicle stability and drug release.

### 2.3. Characterization of niosomes

The characterization of niosomes prepared by thin-film hydration was done using electron microscopy and dynamic light scattering measurements. The techniques provide information on the shape, size, surface charge and distribution of the prepared niosomes.

#### 2.3.1. Transmission electron microscopy (TEM)

A JEM 2100 F JEOL transmission electron microscope was used to analyse the niosome samples in the presence and absence of drugs. The instrument operates at an accelerating voltage of 200 kV, and the samples were assessed under the cryogenic mode due to their biological nature. For sample preparation, the stock solutions of niosomes were diluted to 50 μM and drop cast over formvar-coated 300 mesh copper grids. The samples were air-dried in a desiccator for a minimum of 24 hours before being subjected to analysis.

#### 2.3.2. Scanning electron microscopy (SEM)

The SEM analysis of the niosomes was done on a JEOL, JSM 7600 F scanning electron microscope under cryogenic mode. This enabled observing the surface and size of the niosomes in the hydrated state. The accelerating voltage used for the analysis was 0.1 kV to 30 kV.

#### 2.3.3. Dynamic light scattering (DLS)

The size determination of niosomes was done on a particle size analyser: Litesizer 500, from Anton Paar at 298.15 K and pH 7.4. The hydrodynamic diameter of niosomes in the presence and absence of drugs was analysed based on their Brownian motion. The hydrodynamic diameter (*d*_H_) of niosomes and their Brownian motion is correlated by the Stokes–Einstein equation given below [eqn (1)]1
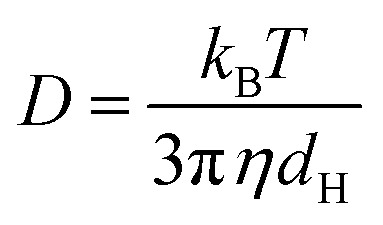


Here *k*_B_ is the Boltzmann constant, *T* is the temperature in Kelvin, *η* is the viscosity of the solvent, and *D* is the translational diffusion coefficient.^39^ The zeta potential (*ζ*) determines the surface charge on the niosomes by calculating its electrophoretic mobility (*μ*_e_), using the equation given below.2
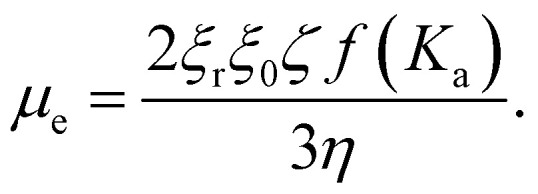


Here *η* is the viscosity of solvent is, *f*(*K*_a_)is Henry's function, *ξ*_r_ and *ξ*_0_ are the relative permittivity and permittivity in vacuum respectively.^40^

### 2.4. Drug encapsulation, release and interaction with HSA

The encapsulation and kinetics of drug release were studied based on thermodynamic parameters, giving an account of the drug that partitions into the empty niosome vesicles upon addition externally. These thermodynamic parameters can also account for the interactions taking place between the drug and the niosome bilayers. The drug partitioning experiments were designed for 25 injections, each releasing a volume of 10 μl into the cell, having a volume of 940 μl. Each injection had a duration of 20 s with an equilibration time of 240 s between two successive injections. The stirring rate in all the experiments was kept at 250 rpm to maintain the overall homogeneity of the solution. For each main experiment, respective dilution experiments were performed and subtracted to assess the final heats of interaction during the partitioning of the drugs in the niosomes.

#### 2.4.1. Drug release

The drug release from Span 60 and Span 60-PEG niosomes was monitored quantitatively (ITC) and qualitatively (spectroscopic methods).

##### 2.4.1.1. Drug release by spectroscopic methods

The drug release was studied using the dialysis method (membrane cut off: 12.5 kDa) and assessed by spectroscopic methods (absorbance and fluorescence), and further compared with the calorimetric observations. The absorbance and fluorescence measurements were performed on a Jasco V-550 double beam spectrophotometer and Cary Eclipse fluorimeter, respectively. The *in vitro* release studies were performed by sealing the drug encapsulated niosomes in a dialysis bag. The dialysis sac was washed and thoroughly cleaned using distilled water, followed by placing the drug encapsulated niosomal suspensions in it. The dialysis bags were then sealed and suspended into 50 ml of PBS buffer maintained at pH 7.4. The solutions were then placed in an incubator at 37.0 ± 0.5 °C and shaking at 100 rpm. The buffer samples were withdrawn at requisite intervals and replaced with fresh buffer solutions to maintain adequate sink conditions. The samples were then diluted and analysed at 234 nm and 610 nm using UV-visible spectroscopy and at 260 nm and 660 nm by fluorescence spectroscopy, respectively, for ketoprofen and mitoxantrone release. The cumulative percentage of drug release was calculated accordingly.

##### 2.4.1.2. Drug release by ITC

To assess the kinetics and determine the thermodynamic parameters related to drug release, isothermal titration experiments were performed on Nano ITC obtained from TA Instruments, USA (injection volume: 250 μl, cell volume: 940 μl). The ITC experiments were designed for a total of 4 injections, each devolving a volume of 50 μl per injection. The cell was filled with PBS buffer, and the syringe was filled with drug encapsulated niosomes. The duration of the injection was kept at 20 s, but the interval between two successive injections was kept at 3600 s. The stirring rate was maintained at 250 rpm to maintain the homogeneity of the solution. The change in heat signatures provides information regarding the release of the encapsulated drugs in the niosomes into the buffer.

#### 2.4.2. Interaction of niosomes with protein

The interaction of niosomes (in the presence and absence of drugs) were assessed with a model protein, human serum albumin (HSA). The interaction studies were performed by loading the syringe of the ITC with drug encapsulated niosomes (S60 + MTX, S60 + KTP, S60-PEG + MTX, S60-PEG + KTP) and titrated into the cell of ITC containing 0.06 mM HSA at 37 °C. The reference cell was filled with buffer solution in all the performed experiments. The interaction of the released drug with HSA was also studied using kinetics protocol developed on ITC. The buffer in the cell was replaced by 0.06 mM HSA. The binding of the released drug from the niosomes with the protein was assessed in terms of thermodynamic signatures. The ITC data were analysed using NanoAnalyse software provided by TA Instruments, USA. The analysis of the data provided the values of partitioning constant (*K*), stoichiometry of binding (*n*), standard molar change in enthalpy 
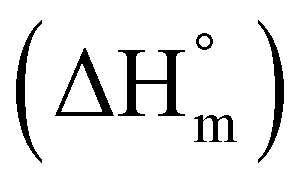
 and standard molar change in entropy 
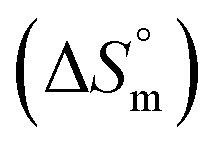
. Through the modified ITC protocol to assess the kinetics of drug release, the associated values of rate constant (*k*) were also determined.

### 2.5. Drug encapsulation efficiency

The non-encapsulated drug was removed from the niosomal formulation by dialysis, followed by the analysis of the entrapment efficiency of niosomes (S60 and S60-PEG). The drug entrapment efficiency was calculated using the following equation.3
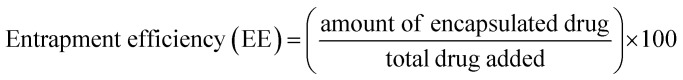


The entrapment efficiency of the niosomes for MTX and KTP was calculated by disrupting the drug containing vesicles with 90% ethanol and analysing the solution by absorbance spectroscopy at 610 nm for MTX and 260 nm for KTP, respectively.^41^

### 2.6. Differential scanning calorimetry (DSC)

The differential scanning calorimetric experiments were performed on a Nano DSC (cell volume of 300 μl) procured from TA Instruments, USA. A pressure of 3 bar and a scanning rate of 1 K min^−1^ were maintained in all the experiments. The thermal unfolding of HSA in the presence and absence of niosomes (with or without incorporated drugs) was done by DSC. The main experiments were rectified by subtracting baselines obtained at the same scan rate by filling both the cells with buffer or buffer with additive(s) (except protein). The baseline corrected DSC profiles were analysed using NanoAnalyse software, and the corresponding values for transition temperature (*T*_m_) and calorimetric enthalpies (Δ*H*_cal_) were determined. The thermal transitions in protein were observed to be irreversible.

### 2.7. Circular dichroism spectroscopy

The circular dichroism experiments were performed on a JASCO CD-180 spectropolarimeter to assess conformational changes occurring in the protein samples upon addition of niosomes, with or without encapsulated drugs. The secondary and tertiary structures of proteins were determined using far UV-CD (260 nm to 180 nm) and near UV-CD (360 nm to 260 nm), respectively. The concentrations of the protein and the path length maintained for far and near UV-CD spectra were 5 μM and 0.2 cm, respectively and 20 μM and 1 cm, respectively. The instrument was purged thoroughly using nitrogen gas during experiments to maintain an inert environment. The molar ellipticity of the protein [*θ*] was calculated using the following equation.4
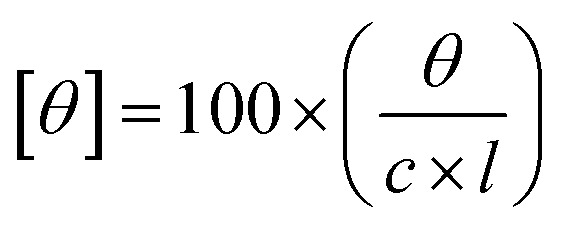


Here ‘*c*’ is concentration of the protein, ‘*θ*’ is specific rotation and ‘*l*’ is path length in cm.^42^

### 2.8. Cell viability

Human triple-negative breast cancer cells, MDA-MB-231, from American Type Culture Collection (ATCC, USA), were maintained in a humidified atmosphere with 5% CO_2_ at 37 °C in Dulbecco's Modified Eagle Medium (DMEM) supplemented with 10% fetal bovine serum (FBS) and 1% (penicillin (100 IU)/streptomycin (0.1 mg ml^−1^) solution). This aggressive cancer cell line was used to explore the biological activity of the vesicles alone and the drug-encapsulated vesicles. The cells were seeded at a density of 5000 cells per well in 96-well, surface-treated plates and allowed to adhere to the surface for 24 h. Subsequently, the medium was replaced with fresh medium containing the desired drug or vesicle of interest (MTX, KTP, S60 + MTX, S60 + KTP, S60-PEG + MTX, S60-PEG + KTP, S60 vesicle, or S60-PEG vesicle) for 72 h. The empty vesicles were prepared from non-ionic surfactants, polyethylene glycol (PEG) and cholesterol. After incubation of the cells for a period of three days, the cell viability was determined using an MTT assay.^43^ Briefly, MTT (3-(4,5-dimethylthiazol-2-yl)-2,5-diphenyl tetrazolium bromide) (0.5 mg ml^−1^ final concentration) was added to each well and incubated for 4 h at 37 °C. The MTT-containing media was then discarded, and 100 μL of dimethylsulfoxide (DMSO) was added to each well and incubated for 10 min. The absorbance of the samples was measured at 570 nm in a Tecan multimode reader (TECAN infinite 200 PRO, Tecan, Switzerland) and the percentage cell viability was calculated as described elsewhere.^44,45^

## 3. Results and discussion

### 3.1. Characterization of niosomes

The characterization of the prepared niosomes in the presence and absence of drugs was done by a combination of electron microscopy (TEM and SEM) and dynamic light scattering experiments (particle size measurements and zeta potential measurements) to assess the structural and morphological changes. The preparation of niosomes was done by the thin-film hydration method, leading to two variants of non-ionic surfactant vesicles with two encapsulated drugs, leading to a total of six systems under analysis.

#### 3.1.1. Transmission electron microscopy

**Fig. 2:**
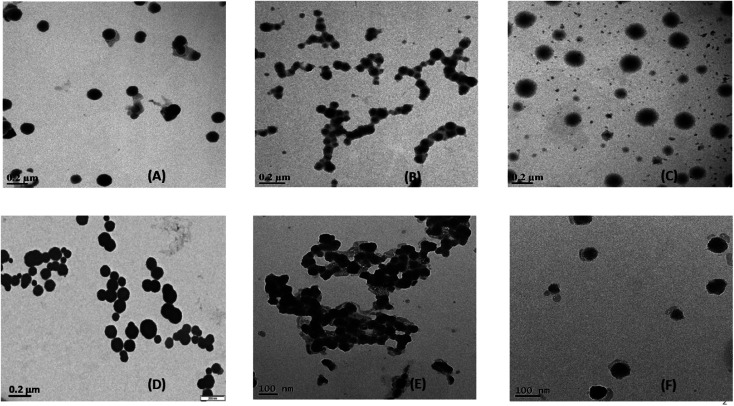
TEM images of (A) S60 niosomes, (B) S60 + MTX niosomes, (C) S60 + KTP niosomes, (D) S60-PEG niosomes, (E) S60-PEG + MTX niosomes and (F) S60-PEG + KTP niosomes.

Fig. 2 represents TEM images of the niosomes in the presence and absence of incorporated drugs. The TEM images for all the systems show uniform distribution of symmetric and spherical niosomes. Fig. 2(A) and (D) show TEM images of S60 and S60-PEG niosomes without any encapsulated drugs. The S60-PEG niosomes have similar shape as S60 niosomes since the PEG molecules conjugate with the cholesterol molecules and wrap around the niosomes.^46^ The size of S60-PEG niosomes is slightly larger than that of S60 niosomes as seen in TEM image and also confirmed by DLS measurements (see Fig. S2†). The size of the niosomes in the presence and absence of drugs as observed from TEM vary from 50–200 nm which is in accordance to the formation of unilamellar niosomes. Fig. 2(B) and (E) represent S60 + MTX and S60-PEG + MTX niosomes showing dark regions indicating the presence of encapsulated mitoxantrone. Fig. 2(C) and (F) show uniform distribution of encapsulated ketoprofen containing niosomes of S60 + KTP and S60-PEG + KTP (indicated by the presence of dark contrast regions inside the niosomes). The TEM images of niosomes show homogeneity in size, shape and distribution of the formed vesicles.

The particle size was analysed using imageJ software for the niosomes (in the presence and absence of drugs). The average particle size was observed to be ranging from 100–250 nm (Table S1†). The morphology of the niosomes was also assessed using scanning electron microscopy which indicated spherical niosomes with varied distribution (Fig. S1†).

#### 3.1.2. Dynamic light scattering experiments

The size and surface charge of the particles were determined in terms of hydrodynamic diameter and zeta potential data obtained from DLS measurements. The addition of PEG during the formation of niosomes and the inclusion of drugs can lead to variation in the particle size and surface charge. The size of the niosomes was analysed using the number percent variation in the size of the particles (Fig. S2† and Table 1). A comparison of hydrodynamic diameter of the niosomes can shed light on the drug encapsulation. In the presence of MTX, particle size of Span 60 niosomes decreases indicating a constriction in the size of the niosomes whereas inclusion of KTP in S60 niosomes shows an almost two-fold increase in size. This can be attributed to the drug encapsulation tendencies of the niosomes, suggesting higher encapsulation of KTP in S60 niosomes as compared to MTX. The presence of PEG in S60 niosomes leads to a slight increase in the particle size, indicating the incorporation of PEG in the bilayers. The PEG molecules are known to conjugate with cholesterol and incorporate in the niosomes. The encapsulation of MTX and KTP in S60-PEG niosomes shows a substantial increase in the hydrodynamic diameter of MTX containing vesicles as compared to KTP containing niosomes. The increase in size is also suggestive of better encapsulation of MTX in PEG containing niosomes as compared to S60 niosomes. KTP, on the other hand shows almost same increase in the hydrodynamic diameter when incorporated in S60 and S60-PEG niosomes indicating similar encapsulation tendencies in both the niosome formulations.

**Table 1:** Hydrodynamic diameter and zeta potential values of niosomes in the presence and absence of drugs

System	Hydrodynamic diameter (*d*_H_)/(nm)	Zeta potential/(mV)
Span 60 niosomes	83.9–116.1	−(45.99 ± 0.96)
(Span 60 + MTX) niosomes	60.7–107.0	−(38.71 ± 1.32)
(Span 60 + KTP) niosomes	116.1–204.6	−(47.90 ± 0.99)
(Span 60 + PEG) niosomes	65.8–160.5	−(44.23 ± 0.97)
(Span 60 + PEG + MTX) niosomes	332.7–460.0	−(39.26 ± 1.02)
(Span 60 + PEG + KTP) niosomes	125.8–221.9	−(48.40 ± 1.10)

The zeta potential of the niosomes is observed to be negative in the presence and absence of drugs with an average value of −(45.99 ± 0.96) mV indicating the presence of anionic polar groups on the outer surface. This negative potential is also imparted due to the presence of a negative charge stabilizer, dicetyl phosphate which prevents the aggregation and clumping of the vesicles. Similar zeta potential values obtained for S60-PEG niosomes indicate that the polar ethylene groups are assimilated around the niosomes structure providing it stability rather than interacting with external components in the solvent environment.^47^ The stabilizer also plays an effect role in S60-PEG niosomes in imparting it stability and charge.

The incorporation of drugs in the niosomes causes minor alteration in zeta potential values. Similar zeta potential values of the drug incorporated niosomes indicate absence of drugs on the outer regions of the niosomes suggesting their effective incorporation into the bilayers. Thus, the drugs added during niosome preparation are incorporated into the bilayers of the niosome, depending on their hydrophilic or hydrophobic characteristics and also functionality. This encapsulation of drugs protects it from the harsh external environment and also helps in sustained release depending on the sink conditions.

### 3.2. Partitioning of drugs in niosomes

As a first step, partitioning of drugs in empty niosomes was explored. ITC experiments were done to study the partitioning of MTX and KTP into niosomes without any preloading. Fig. S3(A) and (B)† represent ITC profiles accompanying titration of 3.5 mM MTX and 7.5 mM KTP into S60 and S60-PEG niosomes at 37 °C. The profiles correspond to titration experiments performed before and after a period of seven days. The experiments were designed to assess the difference in the partitioning of drugs, if niosomes show structural modifications after 7 days. The values of partitioning constant observed for the titration of drugs into S60 and S60-PEG niosomes are of the order of 10^3^ and 10^4^, respectively which indicate weak to moderate encapsulation of the drugs into these self-assemblies. The partitioning of MTX in S60 niosomes show weak two site interaction as analysed by employing independent site models. Other models provided large fitting errors. This indicates that even after a period of 7 days, S60 niosomes offer equivalent partitioning environment. Further insights into the nature of partitioning were assessed through a comparison of enthalpies of interaction. This indicates that even after 7 days, the structure of S60 niosome is not altered to a large extent as reflected by insignificant variation in values of drug partitioning. However, a comparison of the enthalpies of partitioning indicate difference in the nature of interaction between the drug moiety and the niosome structure. The values of enthalpies of partitioning of MTX in the two sites initially are 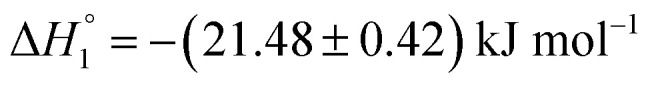 and 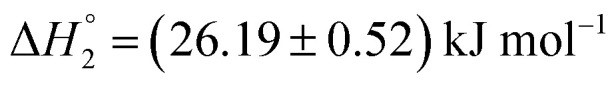, respectively. When assessed after a period of 7 days, these values change to 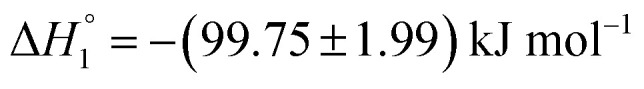 and 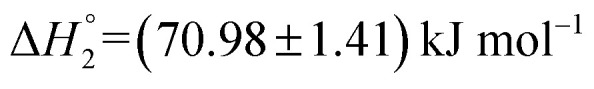. This change in the enthalpic parameters indicates that the minor changes in the structure of the S60 niosomes leads to strengthened exothermic and endothermic interactions between the functional groups present on the drug and the exposed components of the niosome at both the binding events respectively.

Spontaneous processes with an enthalpic penalty are always compensated by entropy gain. The interaction of MTX with S60-PEG niosomes shows a weak partitioning initially and also after a period of seven days. The enthalpies of partitioning do not have appreciable difference (Table 2), suggesting that the presence of PEG in the formulation, provides a structural compactness to the niosome even after period of 7 days. The partitioned drug interacts with the exposed sections of PEG and S60 in a similar manner even after 7 days as indicated by comparison of enthalpic and entropic values. The partitioning process of MTX in S60-PEG also shows enthalpic-entropic compensation. The trends of MTX partitioning in S60 and S60-PEG niosomes also varies as seen in the lower panel of the ITC profiles (Fig. S3†), which indicates a difference in mode of interaction of the drug. The partitioning of KTP was also observed to be quite weak (of the order of 10^3^) with S60 and S60-PEG niosomes initially and also after a period of 7 days (Fig. S4†). The order of partitioning constant remains at 10^3^, indicating a very weak surface interaction between KTP and surface groups of the niosomes.

**Table 2:** The values of partitioning constant (*K*), change in standard molar enthalpy 
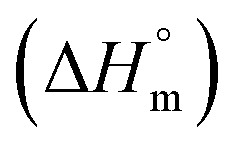
, change in standard molar entropy 
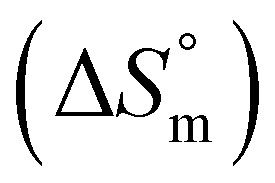
 and stoichiometry (*n*) accompanying the partitioning of mitoxantrone and ketoprofen in empty S60 and S60-PEG niosomes at 37 °C

System	*K*/(M^−1^)	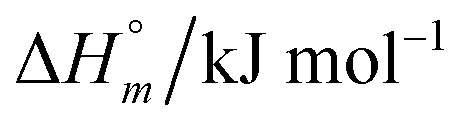	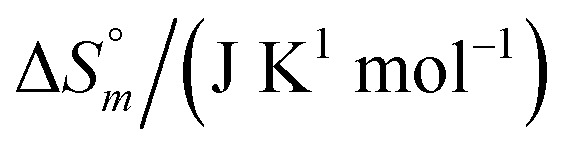	*n*
MTX in S60 (initial fresh preparation)	(0.66 ± 0.02) ×10^4^	−(21.48 ± 0.42)	−(3.9 ± 0.08)	0.10 ± 0.00
	(0.40 ± 0.02) ×10^4^	(26.19 ± 0.52)	(153.5 ± 3.1)	0.10 ± 0.00
MTX in S60 (after 7 days of preparation)	(0.10 ± 0.01) ×10^4^	−(99.75 ± 1.90)	−(264.2 ± 5.2)	0.10 ± 0.00
	(0.17 ± 0.04) ×10^4^	(70.98 ± 1.41)	(291.1 ± 5.8)	0.11 ± 0.00
MTX in S60-PEG (initial fresh preparation)	(1.03 ± 0.01) ×10^3^	−(100.0 ± 2.00)	−(277.7 ± 5.5)	0.10 ± 0.00
	(1.45 ± 0.05) ×10^3^	(78.47 ± 1.56)	(323.8 ± 6.5)	0.12 ± 0.00
MTX in S60-PEG (after 7 days of preparation)	(0.60 ± 0.07) ×10^5^	−(97.31 ± 1.94)	−(222.2 ± 4.4)	0.10 ± 0.00
	(0.65 ± 0.01) ×10^5^	(95.77 ± 1.91)	(401.0 ± 8.0)	0.10 ± 0.00
KTP in S60 (initial fresh preparation)	(1.00 ± 0.02) ×10^3^	−(100.0 ± 0.20)	−(265.0 ± 1.41)	0.10 ± 0.00
	(1.02 ± 0.01) ×10^3^	(93.59 ± 1.87)	(359.6 ± 7.19)	0.11 ± 0.00
KTP in S60 (after 7 days of preparation)	(1.00 ± 0.04) ×10^3^	−(81.93 ± 1.63)	−(206.7 ± 4.1)	0.10 ± 0.00
	(1.04 ± 0.01) ×10^3^	(75.72 ± 1.51)	(301.9 ± 6.0)	0.11 ± 0.00
KTP in S60-PEG (initial fresh preparation)	(1.00 ± 0.02) ×10^3^	−(100.0 ± 1.92)	−(265.0 ± 5.3)	0.10 ± 0.00
	(1.03 ± 0.00) ×10^3^	(94.57 ± 1.89)	(362.6 ± 7.3)	0.11 ± 0.00
KTP in S60-PEG (after 7 days of preparation)	(1.47 ± 0.05) ×10^3^	−(86.36 ± 1.72)	−(229.0 ± 4.6)	0.10 ± 0.00
	(1.50 ± 0.01) ×10^3^	(84.00 ± 1.68)	(342.6 ± 6.9)	0.10 ± 0.00

The partitioning of KTP in S60 and S60-PEG niosomes show an exothermic as well as an endothermic event as reported in Table 2. The interaction of KTP with S60 niosomes is accompanied with enthalpy change of 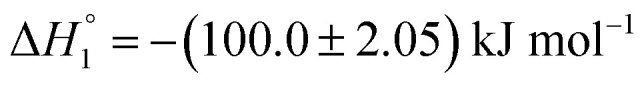 and 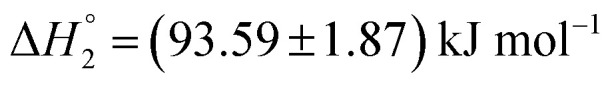 which are similar to those observed for the same system, when assessed after a period of 7 days 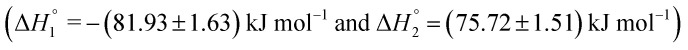. This indicates that the incorporated drug interacts with the outer head regions as well as the inner hydrophobic tails thus indicating an exothermic and endothermic event. The extent of interaction of the drug with S60 niosomes did not increase after a period of 7 days as indicated by the partitioning constants and enthalpic values suggesting the stability and rigidity of the S60 niosomes even after 7 days. The partitioning of KTP in S60-PEG niosomes also follows similar trend as observed with S60 niosomes. The values of partitioning constant are of the order of 10^3^, with either freshly prepared or even after a period of 7 days. The observed thermodynamic signatures also show no variation in the partitioning of drugs during the analysis (Table 2). This indicates that KTP does not partition extensively into the bilayers, leading to its partitioning on the surface of the niosomes. The exothermic enthalpy of partitioning indicates interaction between the polar –COOH groups and the head regions of Span 60 and PEG (in S60-PEG systems) whereas the endothermic enthalpy of interaction could be due to the interaction between the phenyl and hydrophobic components of Span 60 and cholesterol. Thus, the partitioning of the drug KTP on the niosomes occurs near the head regions rather than in the core of the niosomes. The ITC data for drug partitioning of MTX and KTP in the niosomes fits to two independent site models. The interaction of drugs with niosomes can yield exothermic as well as endothermic events depending on the mode and extent of partitioning. The first partitioning event indicates incorporation of the drug near the head regions of the niosome as suggested by the exothermic mode of interaction. The second interaction indicates encapsulation of the drug near the outer palisade region of the bilayer.

### 3.3. Drug encapsulation efficiency

The encapsulation efficiency (see eqn (3)) indicates the amount of drug entrapped in the niosome. It can also be mentioned as the drug retention capacity of the niosomes. During the formation of niosomes, drug is added in the rehydrating medium. After formation the untrapped drug still present in the niosome suspension is removed by dialysis for a period of one hour. The amount of encapsulated drug was calculated by measuring the absorbance of the drug in this dialysate buffer and subtracting it from the absorbance of the stock drug solution. The absorbance values were analysed at 610 nm for MTX and 234 nm for KTP, respectively. The encapsulation efficiency of MTX in S60 niosomes is observed to be (60 ± 5) % whereas for S60-PEG niosomes it is estimated to be (71 ± 5) %. The values of EE of KTP in S60 and S60-PEG niosomes are estimated to be (62 ± 5) % and (65 ± 5) %, respectively. It is observed that the amount of MTX encapsulated in S60-PEG is slightly more as compared to that in S60 niosomes whereas similar amount of KTP is encapsulated in both S60 and S60-PEG niosomes. As mentioned in Section 2.2, 1 mM MTX and 5 mM KTP were incorporated during encapsulation. The EE of drugs in the niosomes is statistically not that different but when we assess using the concentrations of the drug incorporated we observe that S60 incorporated 0.6 mM MTX and 3.1 mM KTP whereas S60 + PEG incorporates 0.7 mM MTX and 3.3 mM KTP. In effect, the amount of KTP incorporated by both the niosomal systems is 5 times more than that of MTX.

### 3.4. Drug release

The drug release from the niosomes was studied using dialysis (assessed by specroscopic methods) and isothermal titration calorimetry.

#### 3.4.1. Dialysis method

**Fig. 3:**
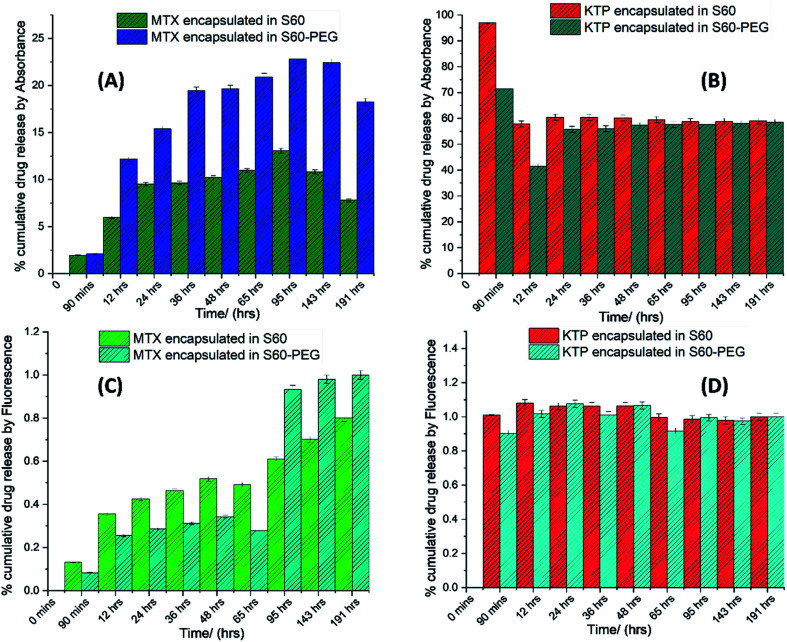
Percent cumulative drug release in (A) MTX incorporated S60 and S60-PEG niosomes, (B) KTP incorporated S60 and S60-PEG niosomes by UV-visible spectroscopy, and (C) MTX incorporated S60 and S60-PEG niosomes, (D) KTP incorporated S60 and S60-PEG niosomes by fluorescence spectroscopy.

After removal of untrapped drug, the niosomes containing MTX and KTP were placed in a dialysis bag and suspended into 30 ml of PBS buffer. The solutions were maintained at 37 °C for a period of 7 days to observe the release of drug from the niosomes and into the buffer. With the passage of time, the drugs would release from the niosomes and accumulate in the buffer. An increase in the concentrations of the drugs in the buffer indicated its amount being released from niosomes with time. The dialysate was removed at fixed time intervals and replaced with fresh buffer to maintain the sink conditions. The dialysate samples were then analysed using UV-visible spectroscopy and fluorescence spectroscopy (Fig. S5† and 3). The release of drug in the buffer led to a considerable change of absorbance in the UV-visible spectra but the intensities were not much pronounced in the fluorescence spectra. This is because a very small amount of the encapsulated drug is released into the buffer which showed considerable absorption but the fluorescence intensities were very low. Hence the normalised data could show a change in the drug release as analysed by absorbance and fluorescence measurements (see Fig. 3).

The representative cumulative plots (Fig. 3) shows the release of mitoxantrone and ketoprofen analysed using UV-visible and fluorescence spectroscopy. The release of MTX from S60 and S60-PEG niosomes is a gradual process which increases with time. It is observed that the concentration of MTX in the buffer increases in a sustained manner with time. This can be related to the concentration and position of the encapsulated drug in the bilayer of the niosomes. In case of KTP incorporated niosomes, a burst release from S60 and S60-PEG niosomes is observed as indicated by sudden increase in absorbance spectra in the range of 250 to 300 nm. An increase in absorbance at 260 nm is due to the release of KTP in the sink buffer. After a burst release (as observed by the analysis of the dialysate), there is a sustained release of the drug in the buffer (Fig. 3). The drawback of this method is that the concentrations of the drug in the buffer are so diluted that the intensities obtained by spectroscopic methods are very less.

The absorbance and fluorescence plots of MTX incorporated niosomes show a distinctive characterization of the extent of drug incorporation in S60 and S60-PEG niosomes. As observed from the plots (Fig. 3(A) and (C)) MTX undergoes a minor burst release followed by a sustained release over the period of analysis. The release of MTX from S60-PEG niosomes is slightly more as compared to the S60 niosomes which is due to the slightly increased drug entrapment in the S60-PEG niosomes. There is a slow increase in the rate of drug release which shows a saturation plateau after 96 hours. Similarly, on assessing the drug release of KTP from S60 and S60-PEG niosomes we initially observe a substantial burst release which reaches saturation and sustained release after a period of 24 h. It is observed that the drug encapsulation of KTP in S60 and S60-PEG niosomes was on average 60% and the drug release observed from both the formulations also follows similar trend. It can be estimated that the incorporation of PEG in the studied niosomes does not affect the encapsulation tendency of KTP. Correlating the structure and partitioning variation of KTP in niosomes it is indiacted that KTP interacts with the Span 60 monomers present in the niosomes more effectively during encapsulation by the interaction of –COOH group of the drug with the hydroxyl groups present on the head regions of S60. Whereas the MTX molecules due to more hydrophobic alkyl groups partition little deeper into the bilayers interacting more with the hydrophilic tail regions of S60, PEG and also slight interactions with cholesterol. This also indicates that the KTP is present near the outer regions of the bilayers leading to burst release compared to that in case of MTX. The qualitative determination of the drug release was done by spectroscopy which was further explored by calorimetry to assess the kinetics and obtain quantitative insights into the release phenomenon.

#### 3.4.2. Kinetics of drug release by isothermal titration calorimetry

**Fig. 4:**
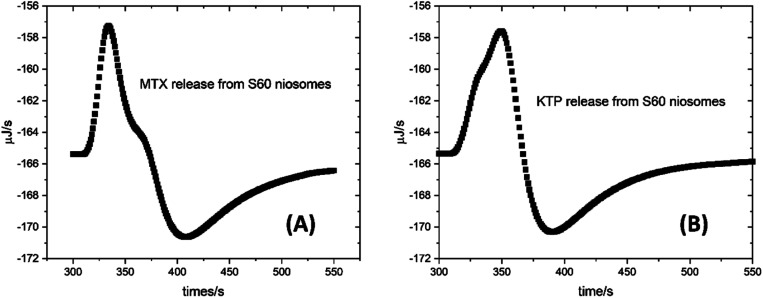
Representative plots of kinetic profiles of drug release of (A) MTX and (B) KTP from S60 niosomes at 37 °C. The plots were fitted to first order kinetics to determine the rate of drug release.

The ITC experiments were performed to quantify and assess the kinetics of drug release. The cumulative release of drug from niosomes provided qualitative understanding and the trends of drug release from niosomes. This can be quantified by assessing the drug release from the profiles obtained by the modified ITC protocol. The qualitative and quantitative drug release parameters can then be correlated to assess the drug encapsulation and release from niosomes. The experiments were performed by the protocol as described in the Section 2.4.1. The data of each injection was analysed according to the first order kinetics. Fig. 4 represents one of the four injections which was analysed to obtain the value of rate constant assuming the release to follow first order kinetics. Table 3 reports the rate of drug release from the S60 and S60-PEG niosomes encapsulating MTX and KTP. The analysis indicates that the rate of drug release initially and after a period of 7 days has minor variations with the latter being slightly higher.

**Table 3:** Kinetics of drug release of 10 mM stock solutions of niosomes (in the absence and presence of drug) in buffer observed at 37 °C by ITC

System	Rate constant (*k*) of drug release in buffer (in s^−1^)
Span 60 niosomes incorporated with MTX (initial drug release)	0.042
Span 60 niosomes incorporated with MTX (after a span of seven days)	0.062
Span 60 niosomes incorporated with KTP (initial drug release)	0.080
Span 60 niosomes incorporated with KTP (after a span of seven days)	0.053
Span 60 and PEG niosomes incorporated with MTX (initial drug release)	0.088
Span 60 and PEG niosomes incorporated with MTX (after a span of seven days)	0.068
Span 60 and PEG niosomes incorporated with KTP (initial drug release)	0.068
Span 60 and PEG niosomes incorporated with KTP (after a span of seven days)	0.066

This is expected since after a time span of 7 days, the structural compactness of the niosomes may decrease leading to slightly enhanced release of drugs. The rate of drug release from S60 niosomes is more in case of KTP (0.080 s^−1^) than MTX (0.042 s^−1^) which is attributed to the higher concentration of the drug being loaded into the niosome. Fig. 4 shows representative plots of drug release from S60 niosomes as monitored by ITC at 37 °C showing the single injection of drug incorporated niosomes into buffer present in the cell of ITC. It shows the change in heat observed due to the release of drug in buffer. The rate of release of MTX is higher in S60-PEG niosomes (0.088 s^−1^) as compared to that from S60 niosomes, which is because of the higher encapsulation of drug in the S60-PEG niosomes. However, after a period of 7 days, though the rate of drug release decreases but it's in a sustained manner. The drug release of KTP from S60-PEG niosomes follow similar kinetics initially and even after a period of 7 days. The above results indicate that the driving force for drug release is mainly diffusion which is concentration dependent. A low value of the rate constant suggests sustained release of the drug for a longer duration under these conditions.

### 3.5. Interaction of drug loaded niosomes with human serum albumin

The interaction of drug loaded niosomes was assessed with human serum albumin (HSA), a major transport protein, to study the interaction of the components of niosomes with the protein. It acts as a carrier protein for the transport of a wide variety of substances in the body and hence its interaction with the niosome components can help in assessing their suitability as drug delivery systems. The interaction of the niosomal formulations with HSA was studied by using a combination of quantitative (ITC) and qualitative (fluorescence spectroscopy) techniques. The quantitative determination of the interaction between niosomes and HSA was assessed by two different methodologies adopted in ITC: the first methodology involved assessing the interactions by titrating the niosomes (in the presence and absence of drugs) with 0.06 mM HSA to determine the thermodynamics of the interaction and second methodology was designed to analyse the kinetics of the binding of the released drug with HSA.

**Fig. 5:**
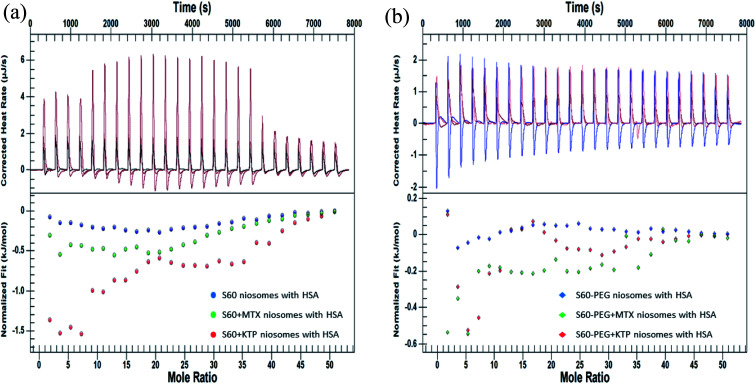
ITC profiles showing the interaction of niosomes (in the presence and absence of drugs) (a) S60, S60 + MTX and S60 + KTP and (b) S60-PEG, S60-PEG + MTX and S60-PEG + KTP with 0.06 mM HSA.

Fig. 5(A) represents the ITC profiles for the interaction of S60 niosomes (in the presence and absence of drugs) with HSA at 37 °C. These ITC profiles have been subtracted by their respective dilution experiments so that these represent actual heats of interaction between the niosome components and the protein.

#### 3.5.1. Quantitative estimation of thermodynamic parameters for interaction of niosomal formulations with HSA

It is observed that S60 niosomes do not show any preferential change in heat signatures and no specific trends when they interact with HSA with an average value of −(0.35 ± 0.01) kJ mol^−1^. Whereas the interaction of MTX incorporated niosomes show a cooperative binding in a sequential mode having enthalpies of interaction as 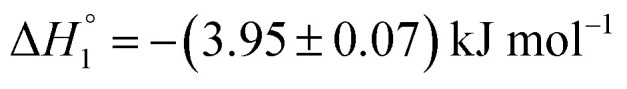 and 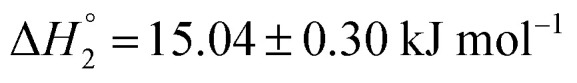. MTX is known to bind at the site III of HSA by hydrophobic, electrostatic and hydrogen bonding interactions. The binding of MTX is known to induce conformational changes in the protein that can facilitate further binding of MTX molecules. The site III has two subdomains III A and III B which can provide binding sites for more than one MTX molecules without causing appreciable conformational alteration in the protein molecule.^48^ The binding of KTP incorporated S60 niosomes leads to a relatively greater change in heat signatures as compared to those with the S60 and S60 + MTX niosomes. This can be assigned to the drug released from S60 + KTP niosomes and its subsequent binding to the protein. KTP incorporated S60 niosomes on interaction with HSA show multiple site binding with two pronounced binding events of the order of 10^3^ (suggesting weak binding of KTP to HSA). Two specific binding events with an enthalpic support of 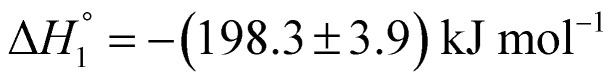 and 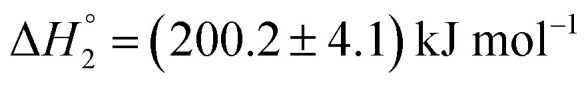 are observed. It is established that ketoprofen binds at the site I and site II of HSA, which is supported by the above observations.^49^ Hence, the drug release of KTP and MTX and interaction of the components of niosome establish that there is no alteration or disruption of protein structure and the drug released is able to bind efficiently to the carrier protein. Both the niosomal formulations show retention of the integrity of protein structure and prevent its structural and conformational modification upon interaction.

Fig. 5(B) shows the interaction of S60-PEG niosomes (in the presence and absence of drugs) with HSA. The trends observed for the interaction of S60-PEG with HSA is similar to that observed with S60 formulations. The S60-PEG niosomes in the absence of drugs do not show pronounced interaction with serum albumin, instead it shows a very minor exothermic enthalpy of interaction approximately quantifying to −(0.15 ± 0.01) kJ mol^−1^ which is even lesser than the interaction of S60 niosomes with HSA. When MTX incorporated S60-PEG niosomes interact with HSA, we observe a cooperative binding process due to the binding of the released MTX at site III of the serum albumin (Fig. 5(A)). It exhibits an exothermic 
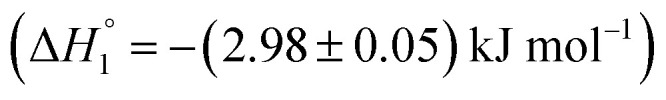
 and an endothermic 
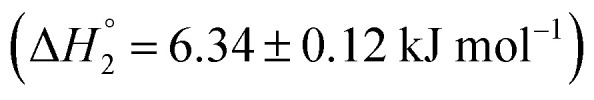
 enthalpy of binding. Here also, cooperative binding suggests minor conformational changes in the protein leading to the binding of drug molecules as observed for MTX released from S60 niosomes. The released drug KTP, from the S60-PEG niosomes show two binding events exhibiting a weak and a relatively moderate affinities of the order 10^3^ and 10^6^. The accompanying enthalpies of interaction 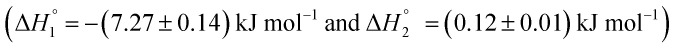 are exothermic and endothermic in nature suggesting the binding of KTP at site I and site II in a different stoichiometric ratio as compared to that of the KTP incorporated S60 niosomes.^46^ An overview of the stoichiometry of binding of the drug molecule *n*_1_ = 0.8 and *n*_2_ = 2.7 indicates that more than one drug molecule is able to bind to the site of HSA, suggesting a slight conformational change in the protein due to the interaction of S60-PEG. The thermodynamic parameters indicate the release of the drug from the niosomes and its subsequent binding to the model protein without creating considerable conformational changes in the protein. Hence, the thermodynamic evaluation suggests niosomes to be viable candidates for drug delivery systems.

#### 3.5.2. Kinetics of release of drugs from niosomes and its binding with HSA

The rate of drug release from the niosomes has been assessed previously in the presence of buffer as the source of sink conditions. Here we have used 0.06 mM HSA as the medium in which drug release is to be observed. These experiments would provide a comparative analysis of drug release when a binding protein is present in the sink medium and also the driving factors for the release of drugs can be assessed. The protocol followed was similar to the experiments for the drug release observed in buffer, where the buffer is replaced with HSA. Table 4 provides the value of rate constant for the drug release from niosomes in the initial phase and after a period of 7 days. The comparison of the kinetic parameters indicates that initially the drug release is less in case of MTX (incorporated in S60 and S60-PEG) which increases when the samples are assessed after a period of 7 days. Whereas the reverse is obtained for the KTP encapsulated niosomes, where we initially observe a high rate of drug release followed by a decline. The drug release from the niosomes in 0.06 mM HSA is probably due to two factors: burst release from KTP encapsulated niosomes and the affinity of the drug towards binding HSA.

**Table 4:** Rate of drug release of 10 mM stock solution of niosomes (in the absence and presence of drug) in 0.06 mM human serum albumin protein observed at 37 °C by ITC

System	Rate of drug release (*k*) (in HSA) in s^−1^
Span 60 niosomes incorporated with MTX (initial drug release)	0.068
Span 60 niosomes incorporated with MTX (after a span of seven days)	0.082
Span 60 niosomes incorporated with KTP (initial drug release)	0.099
Span 60 niosomes incorporated with KTP (after a span of seven days)	0.077
Span 60 and PEG niosomes incorporated with MTX (initial drug release)	0.068
Span 60 and PEG niosomes incorporated with MTX (after a span of seven days)	0.081
Span 60 and PEG niosomes incorporated with KTP (initial drug release)	0.078
Span 60 and PEG niosomes incorporated with KTP (after a span of seven days)	0.066

Since, MTX containing niosomes do not undergo a substantial burst release as compared to KTP encapsulated niosomes, we do not see a pronounced drug release from MTX containing vesicles. Since KTP binds strongly with HSA as compared to MTX, this adds to the driving force for its higher release from the vesicles into the sink conditions. The rates of drug release from niosomes in HSA are slightly higher than the drug release observed in buffer which can be assigned to the affinity of the drug for serum albumin, in addition to diffusion. These results also confirm that the niosomes can show specificity of drug release based on the sink conditions.

#### 3.5.3. Qualitative estimation of drug release from the niosomes in a media containing HSA

The drug release from the niosomes was also assessed using fluorescence spectroscopy to support quantitative insights obtained from ITC. The experimental setup was similar to that used in dialysis experiments with only variations in the sink conditions. Here, the niosomes in the presence and absence of drugs were introduced into clean and pre-washed dialysis bags. These dialysis bags were then suspended into a 30 ml of 7 μM HSA solution prepared in PBS buffer at pH 7.4. The samples were then enclosed in a shaker-incubator and the temperature was maintained at (37 ± 0.5) °C throughout the analysis. Since protein was used as release medium, the analysis was restricted to a period of 48 hours to maintain its structural and conformational integrity. At pre-determined durations, 3 ml of the protein sample was taken and replaced by fresh protein sample so as to maintain same conditions of the released medium. Fig. S6[(A) and (D)]† represents changes in the fluorescence emission upon interaction of niosomes (in the absence of drugs) with HSA. The fluorescence profiles show no quenching indicating that the niosomes or its components do not show any pronounced interaction with the protein, thus not affecting conformational integrity of the latter. Upon analysis of the interaction of MTX incorporated niosomes with HSA (Fig. S6(B) and (E)†), a minor quenching in the emission intensity is observed with the progress of time. It indicates slow and sustained release of the drugs from the niosomes monitored for a period of 48 hours. Since the binding of MTX with HSA is moderate, it does not lead to a pronounced quenching of the protein fluorescence intensity at low concentrations of the released drug from the niosome. In case of drug release from KTP incorporated niosomes, we initially observe a strong burst release as shown by the quenching of the protein thus saturating the protein (see Fig. S6†). After 90 minutes, the proteins solution was replaced with fresh solution of HSA and further analysis of the system showed a sustained release as monitored for a period of 48 hours (Fig. S6(C) and (F)†). As observed earlier in the Section 3.4, here also KTP incorporated niosomes show a burst release as compared to sustained release observed in MTX incorporated niosomes thus supporting calorimetric observations. Fig. S7† shows the consolidated fluorescence profiles representing a comparison of drug release monitored with 7 μM HSA as sink. The profiles clearly show the burst release in case of KTP incorporated niosomes (S60 and S60-PEG) followed by constant rate of drug release with time. Here a slight increase in the release of MTX is also observed from S60-PEG niosomes as compared to S60 niosomes (indicated by comparatively more quenching of the protein fluorescence), thus affirming our previous observations obtained quantitatively from the calorimetric data. The quantitative and qualitative estimation of the effect of the drug encapsulated niosomes and its components also raises a question that how the conformational and thermal stability of the protein are affected in the presence of these components.

The innate quality of a drug delivery system is defined as its ability to sustain and protect the protein structure and functionality. For the same the assessment of thermal and conformational stability of protein during drug binding and effect of the components of drug delivery vehicles on proteins is very crucial. To assess these effects, DSC and CD studies of HSA in the presence and absence of drugs and niosomes were performed. The DSC profiles enable assessment of change in the thermal stability of the model protein HSA in the presence of the niosomal formulations whereas the conformational stability was assessed using CD spectroscopy.

### 3.6. Differential scanning calorimetry

**Fig. 6:**
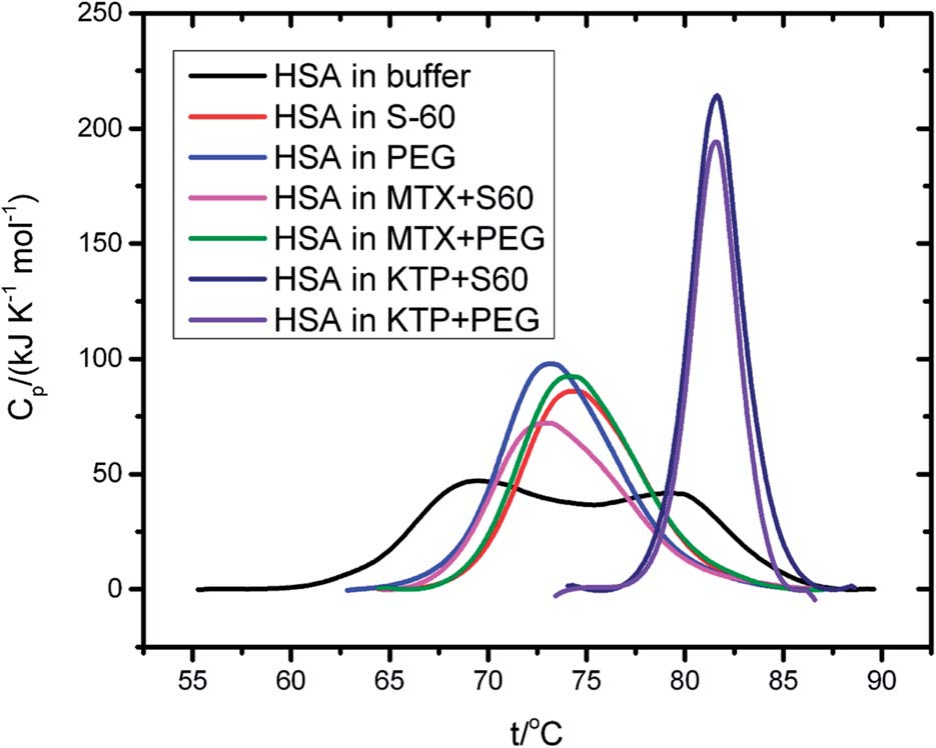
DSC thermograms of 4 mg ml^−1^ human serum albumin in the presence and absence of drug incorporated niosomes in the temperature range of 298.15 K to 363.15 K.

In order to understand the effect of components of niosomes and the incorporated drug on the conformational integrity of the protein HSA, we performed differential scanning calorimetry. Fig. 6 represents DSC thermal unfolding profiles of HSA, in the absence and presence of empty and drug incorporated niosomes. HSA is observed to unfold in a biphasic manner with transition temperatures of (343.1 ± 5.8) K and (352.2 ± 4.8) K and calorimetric enthalpies of 425 ± 6 kJ mol^−1^ and 492 ± 7 kJ mol^−1^, respectively. These results agree with those reported in literature,^50^ though more than two transitions have also been reported for HSA.^51^

In the presence of S60 niosomes (without drugs), a slight thermal stabilisation of the protein at the first transition and a slight decrease in the thermal transition at the second transition was observed with significant alteration in calorimetric enthalpies of unfolding. These results suggest that S60 based niosomes do not affect the thermal stability of the protein significantly though slight conformational change could be induced. Even the MTX incorporated S60 based niosomes provided similar results as that obtained in the absence of drugs. KTP, on the other hand induced a significant thermal stabilisation in the protein when taken together with the S60 niosomes. Here, a thermal stabilisation of 11.2 ± 0.7 K and change in calorimetric enthalpy of 707 ± 11 kJ mol^−1^ are observed.

Inclusion of PEG in niosomes also does not affect the thermal stability of the protein over and above that of S60 and the inclusion of mitoxantrone into the niosomes retains the biphasic thermal unfolding pattern of the protein with similar thermodynamic signatures. Here also KTP into S60 niosomes with PEG led to significant stabilisation of the protein yielding only single endotherm. All these results taken together suggest that the components of niosomes used as drug delivery vehicle do not disrupt the thermal stability and conformational integrity of the protein (Table 5).

**Table 5:** Transition temperature (*T*_m_) and standard molar enthalpy change (Δ_cal_*H*) accompanying the thermal unfolding of 0.06 mM HSA in the presence of drug containing niosomes, at pH = 7.4 and *p* = 0.01 × 10^−5^ Pa in 20 × 10^−3^ mM phosphate buffer. The DSC experiments were done at a fixed pressure of 3.0 × 10^5^ Pa to avoid bubble formation

System	*T*_m_ (1)/K	*T*_m_ (2)/K	Δ_cal_*H* (1)/(kJ mol^−1^)	Δ_cal_*H* (2)/(kJ mol^−1^)
HSA	343.1 ± 5.8	352.2 ± 4.8	425 ± 6	492 ± 7
HSA + S60	346.3 ± 6.9	349.3 ± 6.0	780 ± 12	598 ± 9
HSA + S60 + MTX	345.0 ± 7.0	348.3 ± 4.9	786 ± 12	532 ± 8
HSA + S60 + KTP	354.3 ± 6.5	—	1132 ± 17	—
HSA + S60 + PEG	346.0 ± 5.9	349.9 ± 7.0	728 ± 11	688 ± 10
HSA + S60 + PEG + MTX	346.3 ± 5.7	349.3 ± 7.8	717 ± 11	578 ± 9
HSA + S60 + PEG + KTP	354.6 ± 7.6	—	1349 ± 20	—

### 3.7. Circular dichroism spectroscopy

**Fig. 7:**
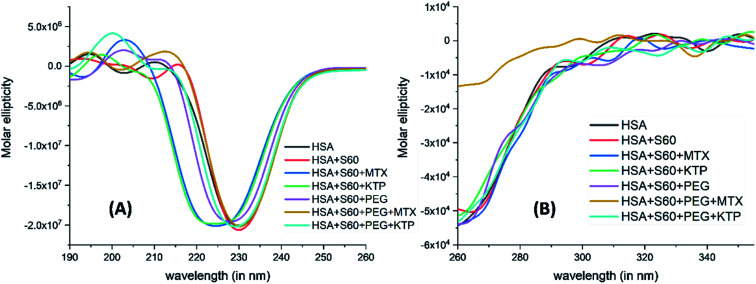
Far UV-CD (A) and near UV-CD (B) spectra of HSA in the absence and presence of drug encapsulated niosomes.

Fig. 7 represents changes in the near UV-CD and far UV-CD regions of the spectra of HSA with and without niosomes or niosome incorporated drugs. The addition of Span 60 niosomes to HSA does not alter the secondary structure of the protein. However, when the MTX encapsulated S60 niosomes are added to the protein, HSA, though the secondary structure of the protein is not disrupted but the entire negative peak shift towards a lower wavelength with an induction of positive band at about 205 nm which is characteristic of beta turns in the protein.

A similar result is observed when the experiment is done in the presence of ketoprofen loaded S60 niosomes. With the modification of S0 niosomes with PEG, the changes in secondary structure of HSA with and without MTX and KTP are insignificant. As seen in the near UV-CD spectra, there is hardly any change in the tertiary structure of the protein with and without the drugs or niosomes. These results suggest that when the S60 based niosomes are used as drug delivery vehicles in the absence or presence of PEG they do not cause any disruption to the conformational stability of the protein. On comparison with DSC results, the niosomes in the presence and absence of MTX convert a broad thermal transition into a sharp one, though the mid-point of transition remains nearly the same. This could indicate slight conformational changes which do not affect the overall protein structure. It is only the presence of KTP which provides an extraordinary thermal stability to the protein as a result of binding but surprisingly no major alterations are seen in the tertiary and secondary structure. All these results taken together establish that the niosomal preparation based on S60 surfactant can act as a safer option to be used in the delivery of drugs.

### 3.8. Inhibition of cancer cell viability

**Fig. 8:**
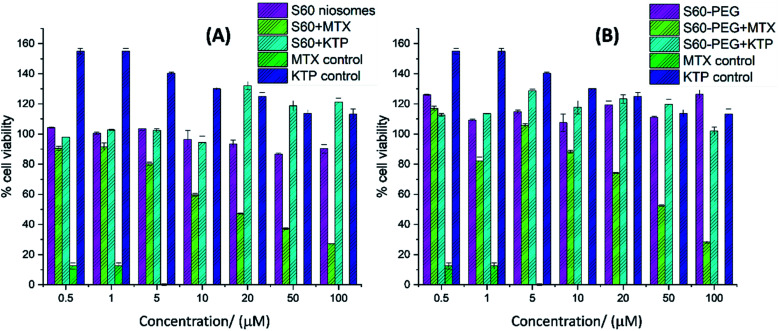
MTT assay for cell viability studies of (A) S60 and (B) S60-PEG niosomes in the absence and presence of MTX and KTP. The cell viability was calculated as a percentage of the ratio of absorbance of treated cells over control. Each point represents the mean value ± SD (*n* = 3).

The viability of MDA-MB-231 cells was studied in the presence of free drug (MTX, KTP), empty niosomes (S60 and S60-PEG), and drug-loaded niosomes. PEG is known to increase the stability and circulation time of the niosomes from a biological point of view and hence the effect of the inclusion of PEG in niosomes can be seen by cell viability studies.^52^ The effect of various substituents on the cell viability as a function of concentration is shown in Fig. 8(A) and (B). It is observed that the empty vesicles (without drug encapsulation) are non-toxic to the cells. They show less inhibition on cell viability confirming that the substituents used in vesicle formation are mostly non-toxic to the cells at the studied concentrations. Among the free drugs tested, mitoxantrone showed considerable toxicity (0.21 ± 0.01 μM) whereas the anti-inflammatory drug ketoprofen enhanced the cell viability.^53^ The drug-loaded niosomes showed the effect of the encapsulated drugs on the cells as they are released with time from the niosomes. The mitoxantrone-containing niosomes showed lesser inhibition of the cell viability as compared to the free drug. This can be assigned to the slow release of the drug from the niosomes as well as the amount of drug being released within the time frame. With the increase in the concentration of mitoxantrone-loaded niosomes, the cell viability decreased. For example, 1 μM, 10 μM, 50 μM and 100 μM S60 + MTX reduced the viability by 9%, 41%, 63%, 73%, respectively and with 1 μM and 10 μM S60 + KTP the viability increased by 2% and for 50 μM and 100 μM S60 + KTP viability increased by 18% and 21%, respectively. S60-PEG vesicles containing MTX reduced the viability considerably. For example, 100 μM of the formulation reduced the cell viability by 72%. KTP incorporated S60-PEG showed an increase in viability (up to ∼20%). In the presence of ketoprofen loaded niosomes, initially the cell viability was not altered, but with increase in concentration of the ketoprofen loaded vesicles cell viability increased.

Although not up to the extent of free MTX-treated cells, the cells treated with niosomes-incorporated MTX reduced cell viability in a concentration-dependent manner. Among the encapsulated ones, the maximum decrease in cell viability was observed in case of MTX-incorporated S60 niosomes followed by MTX-incorporated S60-PEG niosomes. Since the niosomes showed sustained and slow release of drugs, they can be designed to effectively release required doses of drug molecules at designated time intervals.

The statistical analysis was performed using ANOVA calculator including Tukey HSD. The f-ratio was calculated which is the ratio of two variances that are approximately the same when the null hypothesis is true. The f-ratio value is 30.11376 (for data set in Fig. 8(A) and 31.66408 (for data set in Fig. 8(B)) and the *p*-value is <0.0001 and the result is significant at *p* < 0.01.

**Fig. 9:**
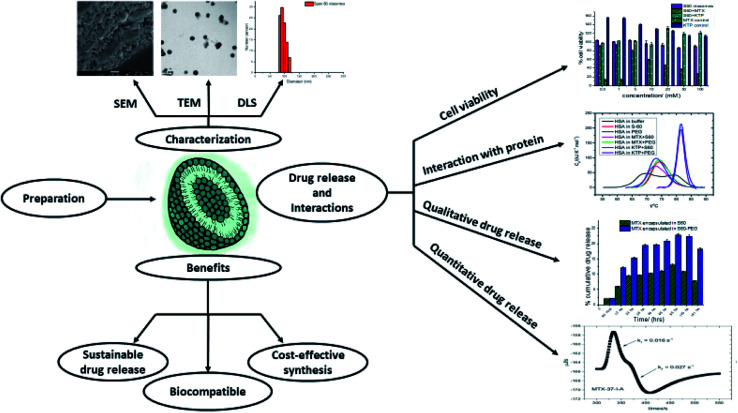
Schematic representation of preparation and characterization of niosomes and assessment of quantitative and qualitative aspects of drug release and its application by biophysical and cell viability assays.

Niosomal systems with suitable choice of its constituent non-ionic surfactants, stabilizers, and other environmental conditions has enabled ese of preparation, characterization, and assessment of not only encapsulation, but also the release of selected drugs qualitatively and quantitatively. Further, biophysical and cell viability assays have permitted understanding efficacy of this drug delivery systems with mechanistic insights (Fig. 9).

## 4. Conclusions

The partitioning and diffusion-controlled release of drugs, mitoxantrone and ketoprofen from the drug encapsulated niosomes of Span 60 and Span 60 with polyethylene glycol have been investigated. The partitioning of the anti-cancer drug, mitoxantrone and the anti-inflammatory drug, ketoprofen in empty niosomes, occurs in the bilayers of the Span 60-based surfactants in order to avoid the surface charge repulsion due to the head groups of the surfactants, cholesterol and stabilizer components present. Similar trends of partitioning of the drugs in the niosomes indicate a structural compactness and rigidity of the drug delivery systems. Weak partitioning, as indicated by ITC results, can be attributed to the surface adsorption of the drugs on the niosomes as a result of weak interactions between the drugs and surface charges on the head groups of niosomes. The release of the drugs from the drug encapsulated niosomes characterized as diffusion controlled followed slow kinetics as monitored by calorimetry and spectroscopy. The systems showed a sustained release for over a period of seven days when analysed at 37 °C at pH 7.4. A novel method of assessing drug release by using ITC was employed which not only enabled heat measurements, but also provided an assessment of kinetics of the release. The sustained release of drugs indicating that these niosome systems are efficient for drug delivery and would help in enhancement of bioavailability of the drugs in the biological system.

The S60 vesicles and S60-PEG based vesicles showed stability and sustained release and can also show stability in morphology and activity for a period of minimum 10 days when stored at 4 °C. The rate of release for both the drugs was comparable with that of ketoprofen to be slightly higher than mitoxantrone which can be attributed to their structure and size. Mitoxantrone, being more hydrophobic, encapsulated deeper into the niosomes which prevents its burst release as compared to that of ketoprofen. The drug release was studied with human serum albumin as a sink to analyse the binding of the released drugs with this carrier protein. The DSC and CD results demonstrate that the components of these niosomal drug delivery vehicles do not affect the conformational and transport ability of HSA. In order to ensure selective elimination of tumour cells, multiple drug delivery systems have been explored, including conjugating potential therapeutic formulations to metal nanoparticles.^54^ The applicability of the niosome systems was also checked on the viability of MDA-MB-231 breast cancer cells. We show efficiency of MTX incorporated vesicles which show effective and sustained release of the drug, that suppressed the viability of the cells. Whereas release of ketoprofen showed enhanced cell proliferation.

Overall, in addition to qualitative assessment, a quantitative understanding of drug partitioning in complex self-assemblies of niosomes has been attempted by employing a novel calorimetric approach. Such studies permit correlations of structure with properties and energetics and enable assessment of effect of components of the drug delivery system on target protein. The developed ITC method has also enabled an assessment of kinetics of drug release and sustainability duration. The current research work highlights the molecular interactions with niosomal systems with insights from qualitative, quantitative and cell biology approaches. The results demonstrate effectiveness of such drug delivery systems and can also guide towards rational drug design.

## Conflicts of interest

There are no conflicts to declare.

## Supplementary Material

RA-011-D1RA06057K-s001
