# Supplementary material for: Mechanistic insights into encapsulation and release of drugs in colloidal niosomal systems: biophysical aspects
Source: RSC Adv. 2021 Oct 29;11(56):35110–26. doi: 10.1039/d1ra06057k (PMC9042874; doi:10.1039/d1ra06057k)
Supplement: RA-011-D1RA06057K-s001 [file RA-011-D1RA06057K-s001.pdf]

Electronic Supplementary Material (ESI) for RSC Advances.
This journal is © The Royal Society of Chemistry 2021

Supplementary Information

Mechanistic insights into encapsulation and release of drugs in colloidal niosomal systems: Biophysical aspects

Eva Judy[a], Manu Lopus[b], Nand Kishore[a,*]

[a]Department of Chemistry, Indian Institute of Technology Bombay, Powai, Mumbai – 400 076, India.

[b]School of Biological Sciences, UM-DAE Centre for Excellence in Basic Sciences, Vidyanagari, Mumbai – 400 098.

Corresponding email address: nandk@iitb.ac.in

Table S1: Analysis of particle size of niosomes (in the presence and absence of drugs) obtained by TEM using Image J software. The average size of the particles are mentioned in nm.

S.No	Area	Mean	Min	Max	Angle	Particle diameter (in nm)
1	1056.604	85.143	58	140	0	165.86
2	1358.491	78.786	40.84	179.849	10.008	212.088
3	981.132	86.206	54.08	144	4.574	154.064
4	943.396	57.286	33.111	103	-12.265	144.588
5	830.189	79.84	35.19	149	10.784	131.321
6	1245.283	17.868	0	64	1.79	196.67
7	1283.019	65.315	33.394	153	1.736	202.81
8	1132.075	83.817	49.241	181	5.906	179.096
9	1320.755	19.486	0	64	0	208.86
10	1094.34	65.241	32	107	0	172.003
11	1094.34	75.78	50.5	142	-2.045	172.112
12	1245.283	38.583	17.781	99	1.79	196.67
13	1245.283	67.488	32.094	189	8.881	198.96
14	1207.547	95.354	51.323	173	9.162	192.893
				Average particle diameter (in nm) = 180.5711		

Figure 2(a) analysis:

Figure 2(b) analysis:

S.No	Area	Mean	Min	Max	Angle	Particle diameter (in nm)
1	1066.667	95.85	45.677	151.113	-30.964	155.492
2	1555.556	57.837	27.242	106	35.538	229.395
3	1955.556	30.471	0	149	13.392	287.827
4	1688.889	70.42	36.012	141	-36.254	248.014
5	1822.222	49.347	6.08	173.378	20.225	269.979
6	1288.889	59.621	14.439	123	39.289	189.502
7	1111.111	72.369	43	112.278	34.992	162.754
8	1644.444	79.33	27.444	149	6.34	241.477
9	2000	52.576	0	110.545	7.765	296.048
10	1600	77.019	35.026	164	10.008	230.169
11	1244.444	83.529	47.42	129	26.565	178.885
12	1911.111	57.525	2.02	179	54.782	277.449
13	1555.556	84.854	25.941	162.118	3.366	227.058
14	1555.556	71.689	36.941	104.471	3.366	227.058
15	1511.111	69.972	21.636	165	37.569	218.683
16	1644.444	89.314	37.926	155.481	33.69	240.37

				Average particle diameter (in nm) = 230.01

Figure 2(c) analysis:

S.No	Area	Mean	Min	Max	Angle	Particle diameter (in nm)
1	2904.69	68.319	16.213	143	2.437	365.949
2	3025.719	70.656	7.143	158	8.297	377.346
3	2481.089	70.683	20.25	132	8.531	314.645
4	2239.032	62.782	16.889	113	3.18	280.479
5	2602.118	53.153	8.333	119	1.364	326.815
6	2057.489	59.907	19.138	147	10.62	253.269
7	1694.402	114.179	73.889	172	6.34	211.328
8	1815.431	80.78	47.372	127.03	15.945	226.531
9	2118.003	76.591	36.503	136	10.305	260.919
10	1694.402	83.422	53.58	130	21.801	209.459
11	2178.517	66.289	21.82	129	25.115	274.923
12	1694.402	82.402	53.272	130	15.068	209.459
13	1996.974	83.433	48.738	131	34.695	245.997
14	1331.316	116.687	68.825	184	41.186	165.386
15	1391.831	104.167	70.998	174	18.435	172.198
16	1149.773	107.006	74.5	144	3.18	140.24
17	1573.374	108.38	75.472	154.424	11.768	190.707
18	1210.287	129.999	107.158	183	15.524	145.326
				Average particle diameter (in nm) = 242.832		

Figure 2(d) analysis:

S.No	Area	Mean	Min	Max	Angle	Particle diameter (in nm)
1	1750	89.545	75.228	166	28.072	240.416
2	1550	89.963	81.14	114	43.668	215.058
3	1650	99.174	82.75	112	29.745	228.035
4	1750	85.374	71.706	97.353	30.379	237.697
5	2300	84.398	66.351	157	44.091	315.04
6	1950	77.471	67.363	126	35.362	268.794
7	1750	77.796	69.519	117	15.255	241.868
8	1800	75.749	64.176	108	30.964	247.386
9	1550	77.215	61.79	180	45	210
10	1900	99.266	80.1	160	-43.919	265.047
11	1600	106.783	96.228	114.809	-13.134	217.83
12	1600	88.086	75.199	131	-38.367	216.449
13	1250	74.335	66.083	83.167	2.386	169.853
14	1550	153.432	86.38	254.967	-64.29	211.896
				Average particle diameter (in nm) = 234.6692		

Figure 2(e) analysis:

S.No	Area	Mean	Min	Max	Angle	Particle diameter (in nm)
1	332	19.434	0	71.684	-12.68	164
2	268	55.151	27.083	77.537	-35.134	132.061
3	288	76.486	55.015	101.381	-21.501	141.873
4	288	47.407	26.857	94	25.115	141.365
5	232	38.65	4.055	67.919	29.249	114.612
6	396	29.307	0.17	68.923	-40.855	195.673
7	196	82.341	37.333	148.812	-38.29	96.83
8	268	132.041	109.596	159.182	19.537	131.575
9	256	110.17	86.245	157.073	-22.479	125.539
10	260	51.996	28.297	86.527	-38.66	128.062
11	260	99.296	65.873	130.24	-20.136	127.812
12	248	110.191	79.601	158	-23.199	121.852
				Average particle diameter (in nm) = 135.1045		

Figure 2(f) analysis:

S.No	Area	Mean	Min	Max	Angle	Particle diameter (in nm)
1	220.134	31.821	0	254.728	48.013	132.26
2	209.396	29.347	1.729	124.675	106.557	126.491
3	163.758	79.647	56.311	115.9	34.216	99.069
4	174.497	98.233	69	160.234	-48.814	104.503
5	144.966	97.246	71.279	148	-55.713	87.255
6	153.02	117.767	88.939	165.204	-37.694	91.108
7	244.295	100.452	53.406	188.462	20.854	147.28
				Average particle diameter (in nm) = 112.5666		

Scanning electron microscopy

The niosomes were assessed using SEM to analyse morphological variations. This technique employs lower magnification than TEM and hence the changes caused by drug incorporation cannot be assessed by this method. Fig. S1 represents the cryo-SEM images of niosomes highlighting their distribution and surface morphology. The vesicular shape is spherical and consists of similar sizes (as observed in TEM) indicating the formation of majorly small unilamellar vesicles (SUV). The niosomes seen in the images are of nanometer size (around 100-

200 nm). The variation in the morphology of niosomes due to incorporation of drugs was observed as seen clearly in Fig S1 (B). The correlation of the size of the niosomes in the presence and absence of drugs was further done by employing DLS.

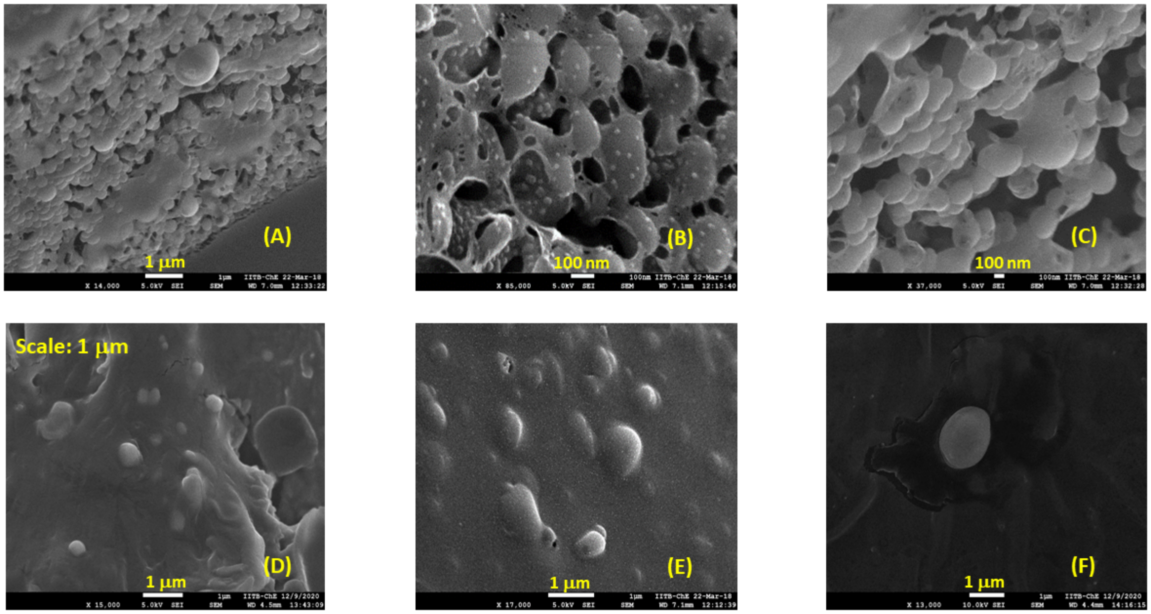

Figure S1. SEM images of (A) S60 niosomes, (B) S60+MTX niosomes, (C) S60+KTP niosomes, (D) S60-PEG niosomes, (E) S60-PEG+MTX niosomes and (F) S60-PEG+KTP niosomes.

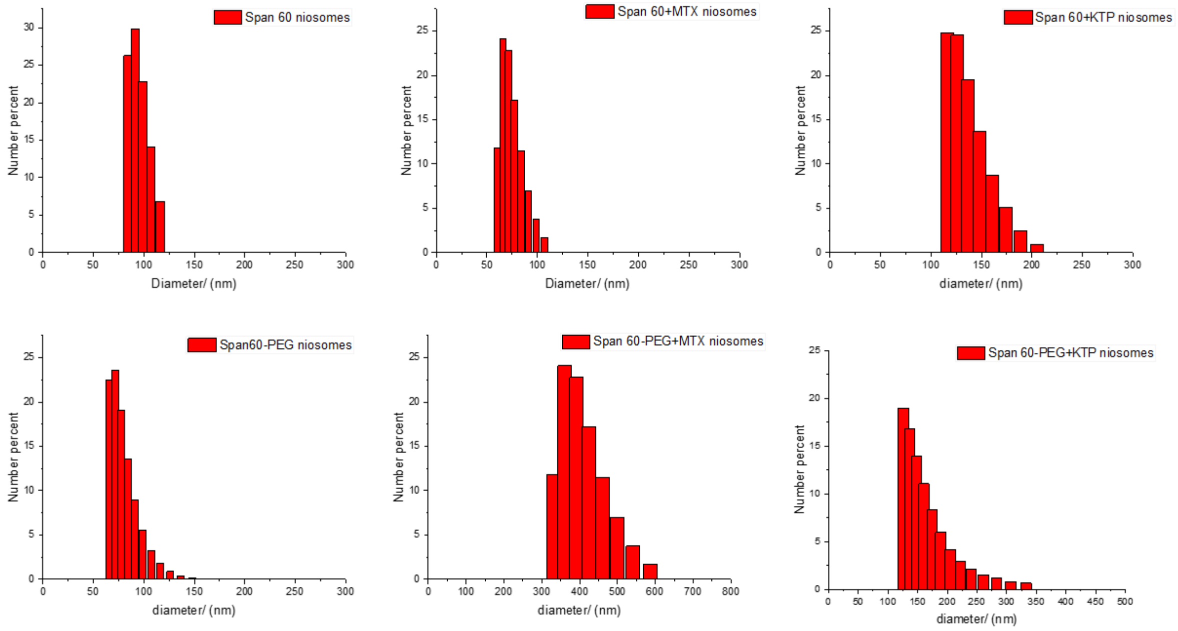

Figure S2. Size distribution of the vesicles represented as change in number percent with diameter of (A) S60 niosomes, (B) S60+MTX niosomes, (C) S60+KTP niosomes, (D) S60-PEG niosomes, (E) S60-PEG+MTX niosomes and (F) S60-PEG+KTP niosomes.

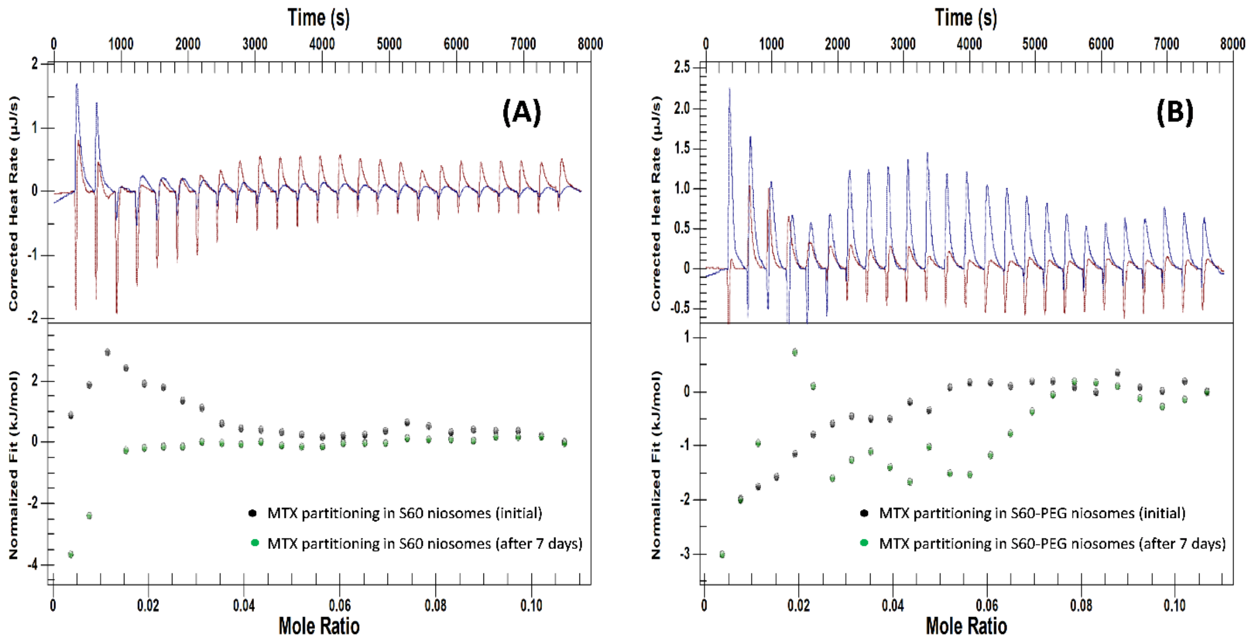

Figure S3. Interaction of MTX with (A) S60 niosomes and (B) S60-PEG niosomes in the initial period after formation of the niosomes (day-1) and after a period of 7 days at 37^0C.

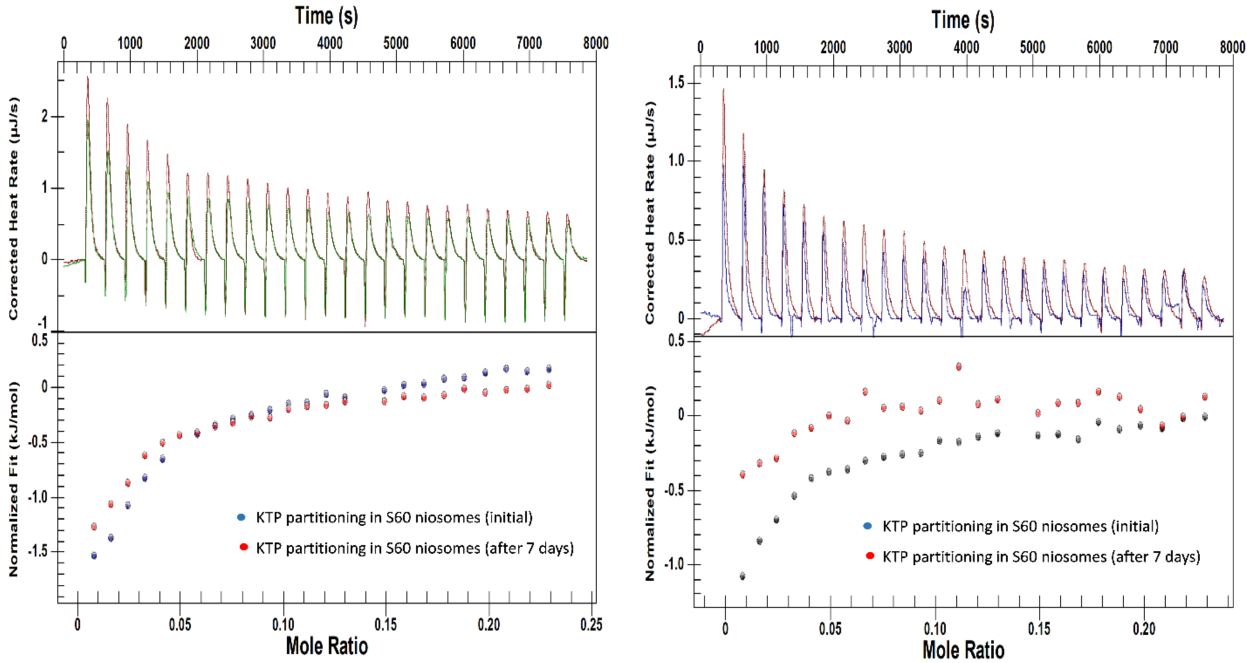

Figure S4. Interaction of KTP with (A) S60 niosomes and (B) S60-PEG niosomes in the initial period after formation of the niosomes (day-1) and after a period of 7 days at 37^{0}C.

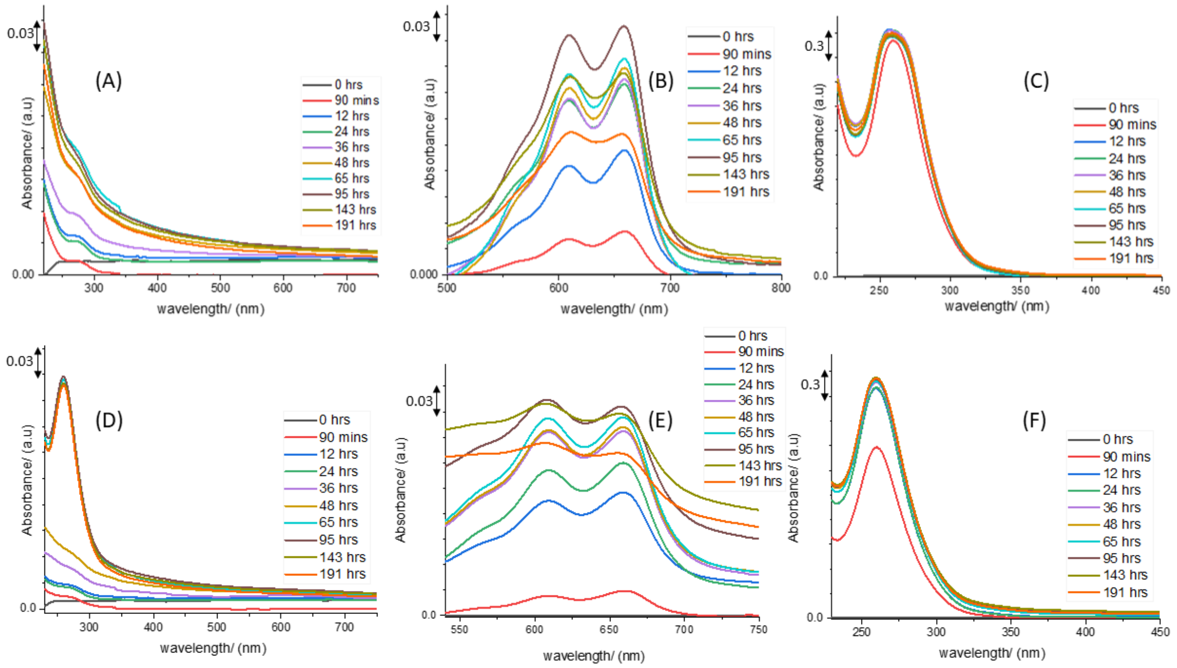

Figure S5. Drug release in S60 and S60-PEG niosomes as monitored by UV-Visible spectroscopy: (A) S60 niosomes (in the absence of drugs), (B) S60+MTX niosomes, (C) S60+KTP niosomes, (D) S60-PEG niosomes, (E) S60-PEG+MTX niosomes and (F) S60-PEG+KTP niosomes.

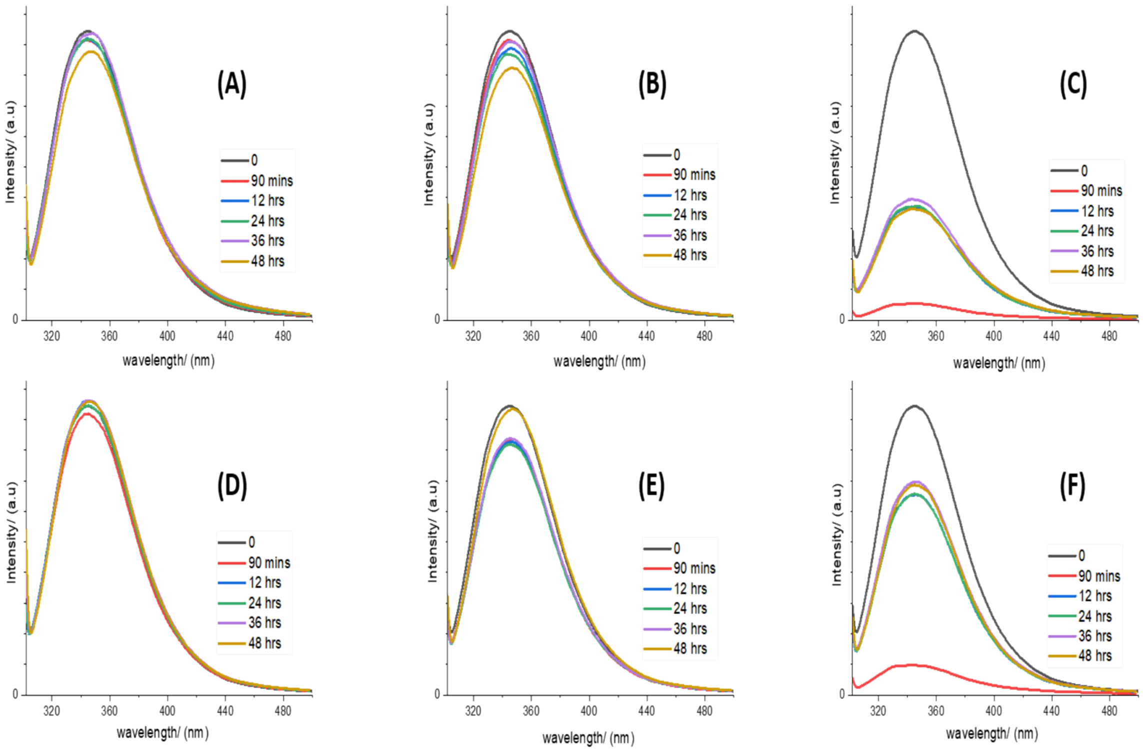

Figure S6. Fluorescence profiles showing the interaction of niosomes (in the presence and absence of drugs) (A) S60, S60+MTX and S60+KTP and (B) S60-PEG, S60-PEG+MTX and S60-PEG+KTP with 0.06 mM HSA as assessed by dialysis methods.

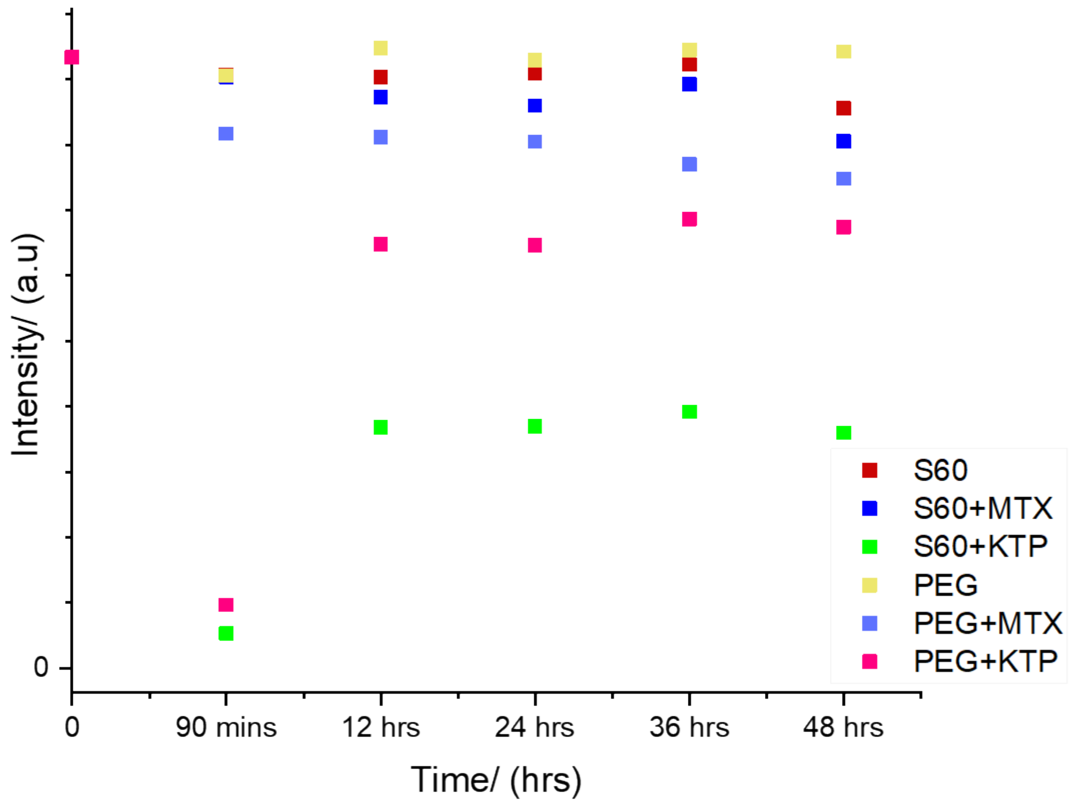

Figure S7. Integrated fluorescence emission profiles showing the interaction of niosomes (in the presence and absence of drugs) with 7 μM HSA assessed by dialysis.
